# Cell Walls of *Saccharomyces cerevisiae* Differentially Modulated Innate Immunity and Glucose Metabolism during Late Systemic Inflammation

**DOI:** 10.1371/journal.pone.0030323

**Published:** 2012-01-17

**Authors:** Bushansingh Baurhoo, Peter Ferket, Chris M. Ashwell, Jean de Oliviera, Xin Zhao

**Affiliations:** 1 Department of Animal Science, McGill University, Quebec, Canada; 2 Department of Poultry Science, North Carolina State University, Raleigh, North Carolina, United States of America; Indian Institute of Science, India

## Abstract

**Background:**

*Salmonella* causes acute systemic inflammation by using its virulence factors to invade the intestinal epithelium. But, prolonged inflammation may provoke severe body catabolism and immunological diseases. *Salmonella* has become more life-threatening due to emergence of multiple-antibiotic resistant strains. Mannose-rich oligosaccharides (MOS) from cells walls of *Saccharomyces cerevisiae* have shown to bind mannose-specific lectin of Gram-negative bacteria including *Salmonella*, and prevent their adherence to intestinal epithelial cells. However, whether MOS may potentially mitigate systemic inflammation is not investigated yet. Moreover, molecular events underlying innate immune responses and metabolic activities during late inflammation, in presence or absence of MOS, are unknown.

**Methods and Principal Findings:**

Using a *Salmonella* LPS-induced systemic inflammation chicken model and microarray analysis, we investigated the effects of MOS and virginiamycin (VIRG, a sub-therapeutic antibiotic) on innate immunity and glucose metabolism during late inflammation. Here, we demonstrate that MOS and VIRG modulated innate immunity and metabolic genes differently. Innate immune responses were principally mediated by intestinal *IL-3*, but not *TNF-α*, *IL-1* or *IL-6*, whereas glucose mobilization occurred through intestinal gluconeogenesis only. MOS inherently induced *IL-3* expression in control hosts. Consequent to LPS challenge, *IL-3* induction in VIRG hosts but not differentially expressed in MOS hosts revealed that MOS counteracted LPS's detrimental inflammatory effects. Metabolic pathways are built to elucidate the mechanisms by which VIRG host's higher energy requirements were met: including gene up-regulations for intestinal gluconeogenesis (*PEPCK*) and liver glycolysis (*ENO2*), and intriguingly liver fatty acid synthesis through *ATP citrate synthase* (*CS*) down-regulation and *ATP citrate lyase* (*ACLY*) and *malic enzyme* (*ME*) up-regulations. However, MOS host's lower energy demands were sufficiently met through TCA citrate-derived energy, as indicated by *CS* up-regulation.

**Conclusions:**

MOS terminated inflammation earlier than VIRG and reduced glucose mobilization, thus representing a novel biological strategy to alleviate *Salmonella*-induced systemic inflammation in human and animal hosts.

## Introduction

*Salmonella* is a leading human food-borne pathogen, worldwide [1]. The pathogen invades the intestinal epithelium by using its specialized Type III secretory systems (T3SS) to cause acute systemic or extra-intestinal inflammation [2]. Indeed, the intestine is the portal of entry through which *Salmonella* triggers systemic infections. However, although life-threatening, treatment of *Salmonella*-induced systemic inflammation has received very little interests in scientific investigations. The disease is frequently caused by consumption of undercooked contaminated poultry meat and meat products [3], which accidentally occur upon exposure to intestine-residing *Salmonella* during chicken processing.

Over decades, low doses of sub-therapeutic antibiotics such as virginiamycin (**VIRG**) have been administered daily in diets of food-producing animals, including poultry, to control intestinal pathogens. Unlike therapeutic antibiotics, sub-therapeutic antibiotics are macromolecules that exert localized bactericidal effects in the intestines only. However, according to the World Health Organization (**WHO**), such practice has debatably been associated with emergence of multiple antibiotic-resistant strains of *Salmonella* [4]. Today, not only has *Salmonella* become more difficult to control in poultry production, but antibiotic treatment of *Salmonella*-induced gastrointestinal and systemic infections has become less successful among hospitalized patients, causing higher death rates [1], [4]. Therefore, the development of natural immuno-modulators that can prevent or treat *Salmonella* infections in both poultry and humans is highly desirable. Evidence exist that mannose-rich oligosaccharides (**MOS**), purified from cells walls of *Saccharomyces cerevisiae*, competitively binds mannose-specific lectin, namely FimH, of Gram-negative bacteria expressing the Type 1 fimbriae, including *Salmonella*, thereby reducing their adherence to mannose-containing glycoprotein receptors on intestinal epithelial cells in humans and chickens [5], [6].

Innate immunity represents the first line of immune defense against invading pathogens in both mammals and avian species. Extracellular Toll-like receptor 4 (TLR-4) of innate immune cells, including macrophages and dendritic cells, recognizes the LPS-endotoxin in outer membranes of Gram-negative bacteria [7]. The engagement of LPS to TLR-4 triggers a cascade of transduction signaling resulting in inflammatory responses characterized by secretion of pro-inflammatory cytokines, including IL-1 and IL-6 that orchestrate pathogen clearance [8]. But, innate immune responses must be regulated exceptionally tightly because high IL-1 and IL-6 levels cause fever, anorexia and bodyweight (**BW**) losses [9], [10], catabolism of skeletal muscles [11]–[13] and adipose tissues [14], and immunological diseases [15] in chickens, rats and humans. Therefore, it is clear that an ideal immune response would be one that can clear pathogens or antigens and be terminated soon after infection. However, despite significant advances in our understanding about inflammatory responses, molecular events of innate immunity and metabolic activities during the period of late inflammation are still not clear. Moreover, whether modulation of intestinal mucosal immunity due to dietary MOS may suppress *Salmonella*-induced systemic inflammation and reduce nutrient mobilization is unknown. The regulatory immune responses between intestinal mucosal and systemic immunity is well recognized [16], [17]. Therefore, considering the human health havoc due to sub-therapeutic antibiotic utilization among food-producing animals, this study evaluated the effects of MOS and sub-therapeutic antibiotics on innate immunity and nutrient metabolism during late *Salmonella* LPS-induced systemic inflammation.

The experiment reported herein, conducted in a chicken model, a frequently utilized biological model in nutrigenomic scientific investigations [18], and using chicken-specific microarrays, reveals that MOS and the VIRG antibiotic differently regulated expressions of genes involved in innate immunity and metabolic pathways during late systemic inflammation. Innate immune responses were principally mediated by intestinal IL-3, but not IL-1 or IL-6. In contrast to VIRG, MOS inherently induced innate immune responses in non-challenged control hosts. Interestingly, however, MOS terminated innate immune responses earlier than VIRG and reduced glucose mobilization.

## Results

LPS induced pathological symptoms, reduced feed intake and BW, and increased liver size in MOS- and VIRG-fed chickens. However, to make a clear distinction between the effects of MOS and VIRG among hosts within the physiological (non-challenged controls) and inflammatory (LPS-challenged) conditions, we relied on microarray results that detailed the coordinately regulated biological mechanisms underlying innate immunity and nutrient metabolism. Tissue-specific RNA extracted from the intestines, liver and skeletal muscles at 24 h post-LPS challenge were analyzed using chicken-specific microarrays. All data files from this experiment have been deposited into the MIAME compliant Gene Expression Omnibus (GEO) database, www.ncbi.nlm.nih.gov/projects/geo (accession no. GSE28959).

### LPS induced clinical symptoms in antibiotic- and MOS-fed chickens

To provoke a systemic inflammatory response, chickens were injected i.p. with a sublethal dose of LPS (3 ml of 100 mg LPS/L). The reaction to LPS is a well-characterized innate immune response [19]. Whether hosts were fed MOS or VIRG, LPS caused symptoms of drowsiness, lethargy, ruffled feathers, moderate diarrhea, starvation and withdrawal from water at 6 h post-LPS injection, thus demonstrating success of our challenge model. These clinical signs of innate immune response were most evident around 8 h after LPS injection. Clinical and behavioral changes due to LPS injection have previously been reported in different animal species, including chickens [9]. No signs of inflammatory responses were observed among non-challenged control hosts. To assess pathological changes further, body temperatures were measured at 0, 2, 4, 6, 8, 12, 24 and 48 h post-LPS injection. Body temperature was similar among all hosts prior (0 h) to LPS challenge (Figure S1 *A*). But, LPS markedly increased body temperatures of MOS- and VIRG-fed hosts after 4 h of LPS challenge, and this effect persisted through 24 h (Figure S1 *A* and *B*). After 48 h, however, all hosts regained their homeostatic state after termination of inflammatory responses (Figure S1 *A* and *B*).

### LPS reduced feed intake and bodyweight gain, and increased liver weight in antibiotic- and MOS-fed chickens

LPS markedly reduced feed intake at 12 h (Figure S2 *A*), but not at 24 and 48 h, post-challenge despite VIRG and MOS supplementations. In addition, LPS's effects in reducing feed consumption and inducing profuse diarrhea at 12 h led to severe loss in BW gain (growth) (Figure S3 *A* and *C*). However, depressed BW gain persisted through 24 and 48 h post-LPS challenge. On the other hand, increased liver weights were observed at 12 and 24 h among LPS-challenged hosts (Figure S4 *A* and *C*). But, the more profound increase in liver weights that occurred after 12 h rather than 24 h (+0.66% vs +0.40% of BW) indicated that higher liver metabolic activities might have occurred at 12 h post-LPS challenge responsive to host's higher energy demands. Moreover, given the similarities in body temperatures, feed intake, and liver weights between LPS-challenge and non-challenge control hosts, it is clear that inflammatory responses were abated at 48 h post-LPS challenge. All of these findings indicated that inflammatory responses were more intense earlier than 24 h post-LPS challenge. Therefore, based on the similarity in feed intake, depressed growth and increased liver weights, but persistence in elevated body temperatures, we concluded that 24 h post-LPS treatment corresponded to late inflammation.

### Main Effects: LPS increased innate immune responses

Our results revealed that LPS significantly increased innate immune responses in intestinal tissues (Table 1) characterized by IL-3 up-regulation, and down-regulation of the gene for zinc finger CCCH-type containing 15 (ZC3H15), which negatively regulates macrophage activation [20]. Additionally, the gene coding for the signal transducer and activator of transcription 2 (STAT2), a signaling pathway that augments macrophage's phagocytic activities against pathogenic bacteria by inducing inducible nitric oxide synthase (iNOS) and lysosomal enzymes [21], was induced in the liver. But, down-regulation of genes were observed for TLR 2 precursor (TLR2-2) that also recognizes and binds LPS [22], gallinacin-1 alpha (Gal-1), a wide spectrum antimicrobial peptide functionally equivalent to human β-defensins [23], and putative CXCR1, an IL-8 receptor that binds the IL-8 chemoattractant expressed by macrophages, monocytes and neutrophils. Indeed, gene expression for 2′–5′-oligoadenylate synthetase A (OAS^*^A), which is involved in viral RNA cleavage inhibiting IFN-γ-mediated viral infections [24], was intestinally down-regulated in the absence of viral infection. However, differential immune gene expressions as observed in the intestines and liver were not detected in muscle tissues.

**Table 1:** Genes identified as differentially expressed due to main LPS effects^1^.

Gene	Gene symbol	Gene ID	Fold change	*P*-value	Gene regulation by LPS^2^
**Intestine**					
***Immune response***					
Interleukin 3	IL-3	474356	1.03	0.00328	+
Toll-like receptor 2 precursor	TLR2-2	769014	0.98	0.00596	−
Zinc finger CCCH-type containing 15	ZC3H15	423992	0.98	0.03575	−
2′–5′ oligoadenylate synthetase A	OAS*A	395908	0.96	0.02579	−
Gallinacin-1 alpha	Gal-1	395841	0.99	0.09106	−
***Metabolism***					
Phosphoenolpyruvate carboxykinase 1	PEPCK	396458	1.05	0.00497	+
Phosphopyruvate hydratase	ENO2	395689	0.94	0.00018	−
3-hydroxy-3-methylglutaryl-CoA reductase	HMGCR	395145	0.95	0.00004	−
***Others***					
Myosin, heavy polypeptide 7, cardiac muscle	MYH7	395350	1.03	0.01938	+
Actin alpha 2, smooth muscle, aorta	ACTA2	423787	0.98	0.04217	−
Myosin light polypeptide 9 regulatory	MYL9	396215	1.04	0.02469	+
**Liver**					
***Immune response***					
Signal transducer and activator of transcription 2	STAT2	6773	1.04	0.00018	+
Putative CXCR1 isoform I and II (IL-8 receptor)	CXCR1	430652	0.97	0.02406	−
***Metabolism***					
Enoyl-CoA hydratase	EHHADH	424877	1.05	0.00017	+
Protein phosphatase 1	PPP1R8	419564	1.03	0.00818	+
Malic enzyme 1	ME	374189	1.03	0.03684	+
5′-AMP-activated protein kinase gamma-2	PRKAG2	420435	1.03	0.00190	+
***Others***					
Deiodinase Type 2	DIO2	373903	0.95	0.00043	−
Iroquois homeobox protein 1	IRX1	374185	1.05	0.00136	+
Potassium voltage-gated channel shaker-related subfamily No 3	KCNA3	404303	1.06	0.00008	+
**Skeletal muscle**					
***Metabolism***					
6-phosphofructokinase (PFK-1)	PFKM	374064	0.95	0.00001	−
***Others***					
Atrial natriuretic factor precursor	NPPA	395765	1.03	0.00005	+

1 Pooled LPS-challenged hosts: MOS+VIRG (antibiotic) groups; The complete raw data have been deposited in the Gene Expression Omnibus (GEO) database, www.ncbi.nlm.nih.gov/projects/geo (accession no. GSE28959).

2 +: up-regulated genes by LPS; −: down-regulated genes by LPS.

### Main Effects: LPS increased glucose mobilization and modified fatty acid metabolism

During acute inflammation, starvation alters host's carbohydrate, protein and fat metabolisms that are orchestrated by synergistically-operated pro-inflammatory cytokines, to meet the body's energy requirements. Subsequent to rapid glucose absorption and oxidative utilization, blood glucose level is maintained by liver glycogenolysis, catabolism of skeletal muscles that generates and mobilizes amino acids for liver gluconeogenesis [12], [13], and catabolism of adipose tissues that triggers liver lipolysis [14].

However, here, we observed that glucose mobilization occurred differently during late inflammation than during immunologically non-challenged conditions. LPS significantly increased intestinal gluconeogenesis by increasing gene expression for phosphoenolpyruvate carboxykinase 1 (PEPCK), a key gluconeogenic enzyme that synthesizes phosphoenolpyruvate from oxaloacetate (Table 1). Evidently, intestinal glycolysis and cholesterol synthesis were repressed as indicated by gene down-regulations for phosphopyruvate hydratase (ENO2), which converts 2-phosphoglycerate into phosphoenolpyruvate, and 3-hydroxy-3-methylglutaryl-CoA reductase (HMGCR), which catalyzes the rate-limiting step in the mevalonate pathway converting 3-hydroxy-3-methylglutaryl-CoA (HMG-CoA) into mevalonate [25], respectively. The high rates of glucose synthesis and glucose trafficking across intestinal epithelial cells enhanced intestinal contractions as indicated by *myosin heavy polypeptide 7 cardiac muscle* (*MYH7*) and *myosin light polypeptide 9 regulatory* (*MYL9*) up-regulations [26]. Contrary to expectations, gluconeogenesis did not occur in the liver. Intriguingly, LPS up-regulated genes coding for malic enzyme (ME), a key enzyme involved in fatty acid synthesis catalyzing the oxidative decarboxylation of malate to pyruvate, NADPH and carbon dioxide [27], and enoyl-CoA hydratase (EHHADH), a key enzyme involved in β-oxidation of fatty acids [28]. Therefore, metabolic energy to support late inflammation was derived mostly from increased fatty acid *de-novo* biosynthesis followed by its catabolism. Unexpectedly, LPS up-regulated gene for 5′-AMP-activated protein kinase gamma 2 (PRKAG2), a low energy sensor that represses acetyl-CoA carboxylase and HMG-CoA reductase to inhibit fatty acid and cholesterol biosynthesis, respectively [29]. In muscle tissues, down-regulation of the gene for 6-phosphofructokinase (PFKM), a regulatory enzyme that converts fructose-6-phosphate into fructose-1,6-biphosphate, suppressed the glycolytic pathway, whereas *atrial natriuretic factor precursor* (*NPPA*) up-regulation caused vasodilatation to increase blood flow [30]. Taken together, all these findings reveal a clear disassociation between glucose mobilization and the biosynthesis and β-oxidation of fatty acids. These discrepancies could be attributed to MOS's immune-stimulatory effects among non-challenged control hosts, as discussed later.

### MOS increased innate immune responses in non-challenged control chickens

Because MOS increases *Salmonella* and *E. coli* clearance of the intestines [5], we thought that MOS may suppress innate immune responses under inflammatory conditions rather than VIRG thereby reducing catabolism of body reserves. However, MOS significantly increased innate immune responses among non-challenged control hosts than VIRG. For instance, several innate immune genes were induced by MOS in the intestines, including *IL-3*, *TLR-3*, *TLR2-2* and *Gal-1* (Table 2). Moreover MOS up-regulated liver genes for putative CXCR1, IL 13 receptor alpha 2 (IL13RA2), which is a specific IL-13 receptor, and CD3 glycoprotein (CD3), which increases T cell activation and signaling of humoral immunity [31]. But, *STAT2* was down-regulated.

**Table 2:** Genes identified as differentially expressed due to MOS in non-challenged control hosts^1^.

Gene	Gene symbol	Gene ID	Fold change	*P*-value	Gene regulation by MOS^2^
**Intestine**					
***Immune response***					
Interleukin 3	IL-3	474356	1.05	2.98E-05	+
Toll-like receptor 3	TLR3	422720	1.04	0.00687	+
Toll-like receptor 2 precursor	TLR2-2	769014	1.03	0.00984	+
2′–5′ oligoadenylate synthetase A	OAS*A	395908	1.15	0.00000	+
Gallinacin-1 alpha	Gal-1	395841	1.03	0.00145	+
***Metabolism***					
Phosphoenolpyruvate carboxykinase 1	PEPCK	396458	1.11	0.00007	+
***Others***					
Myosin, heavy polypeptide 7, cardiac muscle	MYH7	395350	1.04	0.01797	+
Secreted protein acidic cysteine-rich	SPARC	386571	0.94	0.00023	−
Myosin, heavy chain 11, smooth muscle	MYH11	396211	0.87	0.00000	−
Heat shock 10 kDa protein 1	HSPE1	395948	0.90	0.00057	−
Myosin light polypeptide 9 regulatory	MYL9	396215	0.93	0.00117	−
Atrial natriuretic factor precursor	NPPA	395765	1.05	0.00005	+
Iron regulatory protein 1	IRP1	373916	0.92	0.00000	−
**Liver**					
***Immune response***					
Interleukin 13 Receptor Alpha 2	IL13RA2	422219	1.05	0.00002	+
Signal transducer and activator of transcription 2	STAT2	6773	0.97	0.02042	−
Putative CXCR1 isoform I and II (IL-8 receptor)	CXCR1	430652	1.07	0.00018	+
CD3 glycoprotein	CD3D	396518	1.04	0.00010	+
***Metabolism***					
Enoyl-CoA hydratase	EHHADH	424877	0.95	0.01192	−
Protein phosphatase 1	PPP1R8	419564	0.97	0.04035	−
Phosphopyruvate hydratase	ENO2	395689	1.05	0.00362	+
Malic enzyme 1	ME	374189	0.96	0.05583	−
***Others***					
Deiodinase Type 2	DIO2	373903	1.05	0.02708	+
Potassium voltage-gated channel shaker-related subfamily No 3	KCNA3	404303	0.95	0.00649	−
**Skeletal muscle**					
***Others***					
NK2 transcription factor related locus 5	NKX2-5	396073	1.18	0.00000	+
Desmin	DES	395906	0.89	0.00011	−

1 Control hosts: MOS-fed chickens v/s VIRG-fed chickens; The complete raw data have been deposited in the Gene Expression Omnibus (GEO) database, www.ncbi.nlm.nih.gov/projects/geo (accession no. GSE28959).

2 +: up-regulated genes by MOS; −: down-regulated genes by MOS.

### MOS increased glucose mobilization and metabolism in non-challenged control chickens

Augmentation in immune responses by MOS among non-challenged control hosts significantly increased glucose mobilization and metabolism. MOS down-regulated the gene for heat shock protein 1 (HSPE1), which folds and activates newly synthesized linear proteins into functional 3-D proteins [32]; thus, deactivated proteins were increasingly utilized in intestinal gluconeogenesis as mediated by *PEPCK* up-regulation (Table 2). Increased intestinal contractions, in part due to increased glucose absorption across epithelial cells, were mediated by *MYH7* up-regulation but not *MYH11* and *MYL9*. Correspondingly, *NPPA* was up-regulated to ascertain high glucose flux into the liver via the hepatic portal vein. Therefore, to increase the glucose-uptake capacity of liver cells, MOS induced the gene for deiodinase type 2 (DIO2), reported to reduce insulin resistance by increasing intracellular triiodothyronine (T3) levels [33]. Furthermore, down-regulation of *potassium voltage-gated channel shaker-related subfamily 3* (*KCNA3*) significantly increased insulin-stimulated glucose uptake through the GLUT4 glucose transporter, as reported by [34]. As evidenced by *ENO2* up-regulation, high liver glucose increased liver glycolytic activities for energy generation. *ME* and *EHHADH* down-regulations repressed liver fatty acid biosynthesis and β-oxidation, respectively. Therefore, elevated intestinal gluconeogenesis and liver glycolysis were sufficient to meet the host's energy demands.

### LPS mediated innate immune responses differently within MOS and antibiotic chicken groups

So far, we reported inherent immune-stimulatory effects due to independent LPS and MOS treatments. Therefore, simultaneous administration of these treatments was expected to intensify the inflammatory responses. Interestingly, however, our results revealed that MOS counteracted the detrimental effects of LPS on innate immunity. Although we observed intestinal down-regulation of the gene for IL-10, an anti-inflammatory cytokine that causes negative-feedback on secretions of pro-inflammatory cytokines [35], neither *IL-3*, as observed due to LPS (Table 1) treatment alone, nor any other pro-inflammatory cytokines were induced (Table 3). To further support MOS's effect in alleviating inflammatory responses, we observed down-regulations of *TLR2-2*, *TLR-3* and OAS^*^A, and *IL13RA2* and *CD3* in intestinal and liver tissues, respectively. In MOS-fed hosts, innate immune responses after LPS challenge were principally mediated by *ZC3H15* down-regulation that enhanced macrophage activation. In VIRG hosts, however, intestinal *IL-3* and *TLR-3* up-regulations (Table 4) revealed higher LPS-induced inflammatory responses.

**Table 3:** Genes identified as differentially expressed due to LPS within MOS-fed hosts^1^.

Gene	Gene symbol	Gene ID	Fold change	*P*-value	Gene regulation by LPS^2^
**Intestine**					
***Immune response***					
Interleukin 10	IL-10	428264	0.97	0.00343	−
Toll-like receptor 3	TLR3	422720	0.95	0.00024	−
Toll-like receptor 2 precursor	TLR2 -2	769014	0.96	0.00036	−
Zinc finger CCCH-type containing 15	ZC3H15	423992	0.95	0.00012	−
2′–5′-oligoadenylate synthetase A	OAS*A	395908	0.82	0.00000	−
Gallinacin-1 alpha	Gal-1	395841	0.96	0.00013	−
***Metabolism***					
Phosphopyruvate hydratase	ENO2	395689	0.93	0.00434	−
3-hydroxy-3-methylglutaryl-CoA reductase	HMGCR	395145	0.95	0.00338	−
***Others***					
Secreted protein acidic cysteine-rich	SPARC	386571	1.04	0.01425	+
Myosin, heavy chain 11, smooth muscle	MYH11	396211	1.10	0.00015	+
Actin alpha 2, smooth muscle, aorta	ACTA2	423787	0.94	0.00006	−
Myosin light polypeptide 9 regulatory	MYL9	396215	1.10	0.00006	+
Iron regulatory protein 1	IRP1	373916	1.04	0.01284	+
**Liver**					
***Immune response***					
Interleukin 13 Receptor Alpha 2	IL13RA2	422219	0.97	0.00431	−
Signal transducer and activator of transcription 2	STAT2	6773	1.07	0.00000	+
Putative CXCR1 isoform I and II (IL-8 receptor)	CXCR1	430652	0.93	0.00007	−
CD3 glycoprotein	CD3D	396518	0.98	0.04308	−
***Metabolism***					
UDP Glucose Pyrophosphorylase 2	UGP2	373900	1.00	0.00492	−
Enoyl-CoA hydratase	EHHADH	424877	1.10	0.00000	+
Protein phosphatase 1	PPP1R8	419564	1.06	0.00017	+
Phosphopyruvate hydratase	ENO2	395689	0.91	0.00000	−
ATP citrate synthase	CS	1431	1.07	0.00092	+
5′-AMP-activated protein kinase gamma-2	PRKAG2	420435	1.07	0.00001	+
***Others***					
Deiodinase Type 2	DIO2	373903	0.94	0.00134	−
Iroquois homeobox protein 1	IRX1	374185	1.09	0.00001	+
Endothelial PAS domain protein 1	EPAS1	395596	1.10	0.00033	+
Potassium voltage-gated channel shaker-related subfamily No 3	KCNA3	404303	1.13	0.00000	+
**Skeletal muscle**					
***Metabolism***					
6-phosphofructokinase (PFK-1)	PFKM	374064	0.95	0.00058	−
***Others***					
Atrial natriuretic factor precursor	NPPA	395765	1.03	0.00417	+

1 Hosts fed MOS: LPS-challenged v/s non-challenged controls; The complete raw data have been deposited in the Gene Expression Omnibus (GEO) database, www.ncbi.nlm.nih.gov/projects/geo (accession no. GSE28959).

2 +: up-regulated genes by LPS; −: down-regulated genes by LPS.

**Table 4:** Genes identified as differentially expressed due to LPS within antibiotic-fed hosts^1^.

Gene	Gene symbol	Gene ID	Fold change	*P*-value	Gene regulation by LPS^2^
**Intestine**					
***Immune response***					
Interleukin 3	IL-3	474356	1.04	0.00404	+
Toll-like receptor 3	TLR3	422720	1.03	0.01264	+
2′–5′-oligoadenylate synthetase A	OAS*A	395908	1.13	0.00000	+
***Metabolism***					
Phosphoenolpyruvate carboxykinase 1	PEPCK	396458	1.12	0.00002	+
Phosphopyruvate hydratase	ENO2	395689	0.94	0.01255	−
3-hydroxy-3-methylglutaryl-CoA reductase	HMGCR	395145	0.95	0.00369	−
***Others***					
Myosin, heavy polypeptide 7, cardiac muscle	MYH7	395350	1.03	0.04746	+
Myosin, heavy chain 11, smooth muscle	MYH11	396211	0.92	0.00094	−
Atrial natriuretic factor precursor	NPPA	395765	1.03	0.00679	+
Iron regulatory protein 1	IRP1	373916	0.96	0.02641	−
**Liver**					
***Metabolism***					
ATP citrate lyase	ACLY	395373	1.04	0.04584	+
Phosphopyruvate hydratase	ENO2	395689	1.05	0.00869	+
Malic enzyme 1	ME	374189	1.06	0.01385	+
ATP citrate synthase	CS	1431	0.96	0.03024	−
**Skeletal muscle**					
***Metabolism***					
6-phosphofructokinase (PFK-1)	PFKM	374064	0.95	0.00246	−
***Others***					
Atrial natriuretic factor precursor	NPPA	395765	1.03	0.00416	+

1 Hosts fed antibiotic (VIRG): LPS-challenged v/s non-challenged controls; The complete raw data have been deposited in the Gene Expression Omnibus (GEO) database, www.ncbi.nlm.nih.gov/projects/geo (accession no. GSE28959).

2 +: up-regulated genes by LPS; −: down-regulated genes by LPS.

### LPS mobilized energy differently within MOS and antibiotic chicken groups

To further evidence that MOS reduced innate immune responses, here we report that LPS failed to induce gluconeogenesis or any other major nutrient mobilization processes among MOS-fed hosts (Table 3). Previously, however, we observed increased intestinal gluconeogenesis due to LPS in the absence of MOS (Table 1). These results, together with reduced liver glucose uptake mediated by *DIO2* down-regulation and *KCNA3* up-regulation, led us to believe that liver glucose levels were sufficiently high to meet the host's energy demands. However, reduced glycolytic activities, due to *ENO2* down-regulation, demonstrated that liver glucose levels were abnormally low. Intriguingly, despite reduced glycolysis, we observed up-regulation of the gene for ATP citrate synthase (CS), which catalyzes citrate synthesis from acetyl Co-A and oxaloactetate. Given that citrate is the key regulatory substrate of the TCA cycle, our results indicated that host's energy demands were likely met mainly via the TCA cycle. We also observed that glucose utilization for energy caused down-regulation of *UDP glucose pyrophosphorylase 2* (*UGP2*), which reduces liver glycogen synthesis [36], whereas *PRKAG2* up-regulation repressed fatty acid and cholesterol biosynthesis. Finally, MOS down-regulated the gene for α-actin 2 in intestinal smooth muscles (ACTA2) that reduced intestinal contractions [37], whereas *MYH11* and *MYL9* were up-regulated. Down-regulation of *HMGCR* repressed intestinal cholesterol synthesis.

**Figure 1:**
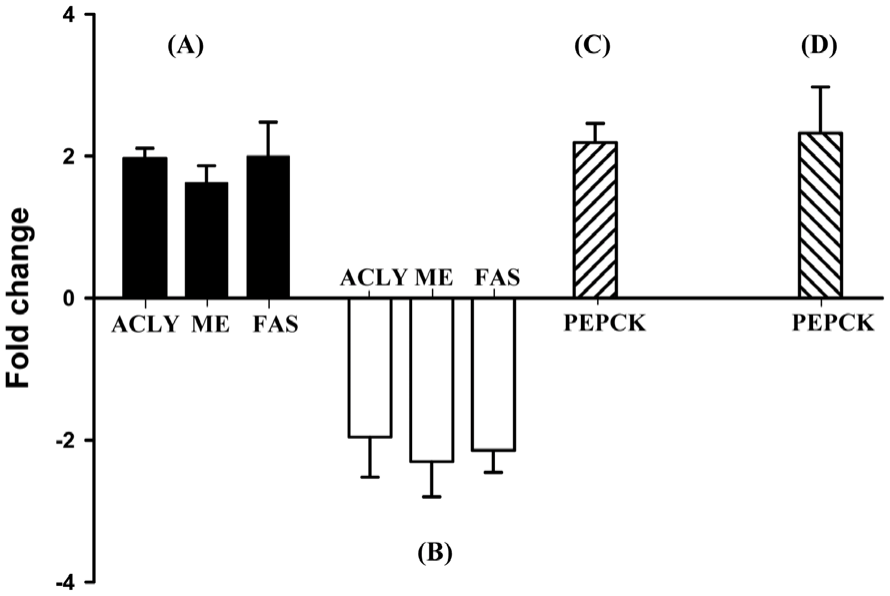
RT-qPCR validation of microarray data. LPS up-regulated liver *ACLY*, *ME* and *FAS* among VIRG-fed hosts (*A*). But, LPS down-regulated these genes in MOS- than VIRG-fed host (*B*). Intestinal *PEPCK* was up-regulated among control hosts fed MOS (*C*), and by LPS among VIRG-fed hosts (*D*). Data are presented as mean ± SEM (*n* = 6). *, *P*<0.01 by Scheffe's *t* test.

Contradictorily, despite VIRG supplementation, LPS challenge profoundly increased gluconeogenesis, as indicated by *PEPCK* up-regulation in the intestines (Table 4). Evidently, *MYH7* was up-regulated whereas *ENO2* and *HMGCR* were down-regulated to suppress intestinal glycolysis and cholesterol synthesis, respectively. But, high glucose influx into the liver increased glycolytic activities through *ENO2* up-regulation. Therefore, glucose metabolites were most increasingly utilized in TCA cycle for energy generation. Surprisingly, *CS* was down-regulated and citrate was instead utilized in fatty acid biosynthesis, as demonstrated by increased expressions of genes for ATP citrate lyase (ACLY), which catalyzes citrate cleavage into acetyl Co-A and oxaloacetate [38], [39], ME and fatty acid synthetase (FAS; as shown in Figure 1A). *PFKM* down-regulation and *NPPA* up-regulation occurred in muscle tissues of both MOS- and VIRG-fed hosts.

### MOS increased innate immune responses in LPS-challenged chickens

In comparisons to LPS-challenged hosts fed VIRG, the additive immune stimulatory effects of LPS and MOS significantly increased innate immune responses as demonstrated by intestinal *IL-3* up-regulation, and down-regulation of *IL-10* and *ZC3H15* (Table 5). But, *TLR-3*, the corresponding receptor to IL-3, was repressed in the intestines. *STAT2* was up-regulated in the liver.

**Table 5:** Genes identified as differentially expressed due to LPS between MOS- and antibiotic-fed hosts^1^.

Gene	Gene symbol	Gene ID	Fold change	*P*-value	Gene regulation by MOS^2^
**Intestine**					
***Immune response***					
Interleukin 3	IL-3	474356	1.03	0.00871	+
Interleukin 10	IL-10	428264	0.96	0.00028	−
Toll-like receptor 3	TLR3	422720	0.96	0.00053	−
Zinc finger CCCH-type containing 15	ZC3H15	423992	0.96	0.00100	−
2′–5′-oligoadenylate synthetase A	OAS*A	395908	0.84	0.00000	−
Gallinacin-1 alpha	Gal-1	395841	0.98	0.03227	−
***Others***					
Actin alpha 2 smooth muscle aorta	ACTA2	423787	0.94	0.00017	−
Heat shock 10 kDa protein 1	HSPE1	395948	0.92	0.00756	−
**Liver**					
***Immune response***					
Signal transducer and activator of transcription 2	STAT2	6773	1.03	0.02089	+
***Metabolism***					
UDP Glucose Pyrophosphorylase 2	UGP2	373900	0.92	0.00009	−
ATP citrate lyase	ACLY	395373	0.93	0.00017	−
Phosphopyruvate hydratase	ENO2	395689	0.91	0.00000	−
Malic enzyme 1	ME1	374189	0.92	0.00012	−
ATP citrate synthase	CS	1431	1.11	0.00000	+
5′-AMP-activated protein kinase gamma-2	PRKAG2	420435	1.05	0.00290	+
***Others***					
Iroquois homeobox protein 1	IRX1	374185	1.06	0.00268	+
Endothelial PAS domain protein 1	EPAS1	395596	1.09	0.00158	+
Potassium voltage-gated channel shaker-related subfamily No 3	KCNA3	404303	1.07	0.00045	+
**Skeletal muscle**					
***Others***					
NK2 transcription factor related locus 5	NKX2-5	396073	1.14	0.00000	+

1 LPS-challenged hosts: MOS-fed chickens v/s VIRG-fed chickens; The complete raw data have been deposited in the Gene Expression Omnibus (GEO) database, www.ncbi.nlm.nih.gov/projects/geo (accession no. GSE28959).

2 +: up-regulated genes by MOS; −: down-regulated genes by MOS.

### MOS mobilized energy differently than antibiotic in LPS-challenged chickens

Despite increased immune responses in LPS-challenged hosts fed MOS, these chickens faced no detrimental nutrient mobilization processes when compared to LPS-injected hosts fed VIRG. Intestinal gluconeogenesis did not occur although newly-synthesized proteins were deactivated by *HSPE1* down-regulation (Table 5). Again, our results about *CS* up-regulation demonstrate that energy was essentially derived from increasingly synthesized citrate in the liver. Under the effects of LPS, MOS repressed glycogen synthesis by down-regulating *UGP2*; glycolysis by down-regulating *ENO2*; fatty acids biosynthesis by down-regulating *ACLY*, *ME* and *FAS* (Table 5 and Figure 1B); and cholesterol biosynthesis by up-regulating *PRKAG2*. Liver *KCNA3* was up-regulated in the absence of high glucose influx. Indeed, these findings are very similar to those observed when comparing MOS-fed hosts in the LPS-challenged and non-challenged control groups.

### Real-time quantitative PCR and liver metabolite measurements

**Figure 2:**
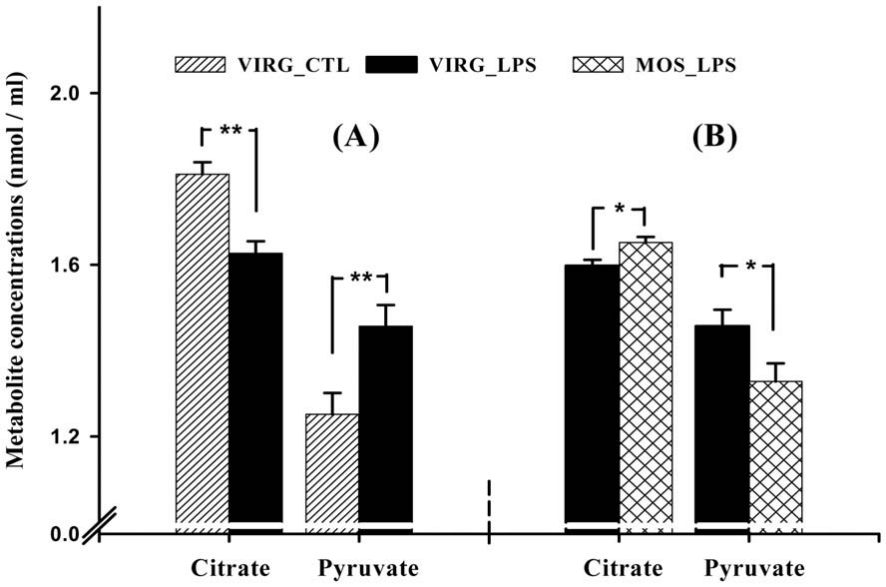
Concentrations of liver metabolites. Among VIRG-fed hosts, LPS reduced liver citrate, but increased pyruvate levels (*A*). However, higher citrate and lower pyruvate levels were observed in liver of LPS-challenged hosts fed MOS than VIRG (*B*). Data are presented as mean ± SEM (*n* = 6). *, *P*<0.05, **, *P*<0.01 by Scheffe's *t* test.

To confirm our microarray data, we performed quantitative RT-qPCR analysis on three differentially expressed genes, and measured concentrations of specific liver metabolites. Figure 1 and Tables 2, 4 and 5 show that *PEPCK*, *ACLY* and *ME* expression patterns correlated strongly with microarray results. Moreover liver citrate and pyruvate levels were in agreement with gene expression results (Figure 2). *FAS*, not present on the array utilized in this study, expression (Figure 1) was determined by RT-qPCR. All RT-qPCR efficiency (E) values were in between 93 to 100%.

## Discussion

Molecular events underlying late inflammation and subsequently nutrient mobilization, in response to pathogens or antigens, are still not clear. Interestingly, at 24 h post-LPS challenge, microarray results revealed that innate immune responses were principally mediated by IL-3, a pro-inflammatory cytokine that has received little scientific investigations, together with other innate immune mediators (Table 1). Few studies reported IL-3 as playing key roles in linking innate and adaptive immunity. IL-3 is critical for the differentiation of monocytes into dendritic cells, and contributes in proliferation and survival of dendritic cells [40]; dendritic cells are involved in Th cell response. While *IL-1* and *IL-6* were consistently up-regulated during intense inflammatory responses in poultry [41] and mice [42], here we report that these pro-inflammatory cytokines were not differentially expressed during late inflammation. These results evidenced that inflammation is a time-dependent biological immune reaction, regulated by different immune mediators. Most interestingly, our results revealed that dietary MOS modulated innate immune responses and nutrient metabolisms differently than VIRG.

**Figure 3:**
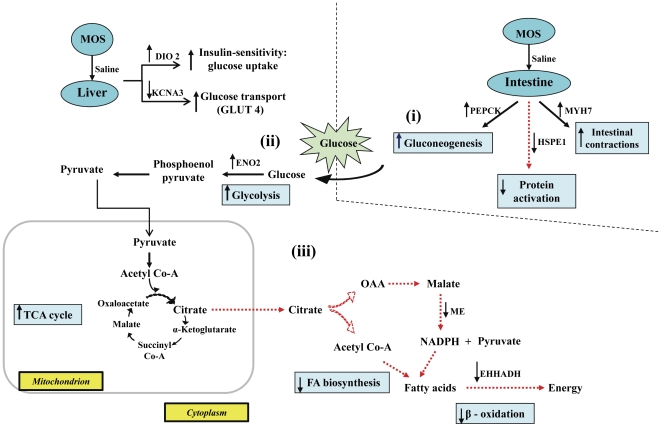
Schematic illustration of the effects of MOS versus VIRG on glucose metabolism in control hosts. (i) MOS increased intestinal gluconeogenesis by up-regulating *PEPCK*; (ii) the high glucose influx into the liver was rapidly metabolized by glycolysis as mediated by *ENO2* up-regulation; (iii) TCA-derived energy from glycolytic substrates, down-regulated *ME* and *EHHADH* which reduced fatty acid synthesis and β-oxidation, respectively.

Our finding that MOS increased immune responses of non-challenged control hosts, but here principally mediated by intestinal IL-3, is consistent with published data [43]-[45], thereby revealing its inherent immune-stimulatory properties. Although the mechanism by which MOS inherently stimulates immunity is unclear, it may be associated with the antigenic properties of yeast cell walls. In contrast, VIRG did not confer such immune stimulatory effects because antibiotics lack antigenic properties. During immune stimulation, an energy-demanding biological process, and the consequential reduction in feed intake, the host's metabolic activities were coordinately regulated to increase energy availability for metabolism. Liver gluconeogenesis, involving muscle catabolism, usually occurs during intense inflammation and starvation [12]. However, here, we observed that gluconeogenesis occurred only in the intestines. Additionally, during the process of glucose synthesis, the preferential utilization of amino acids from ingested feed spared skeletal muscle catabolism. Glucose, mobilized to the liver, was then rapidly metabolized via increased glycolytic activities to meet host's elevated energy demands during the inflammatory response (summarized in Figure 3). Nevertheless, as previously reported [6], [46], MOS's immune-stimulatory effects did not profoundly mobilize glucose and had no detrimental effects on feed intake or growth.

Both LPS and MOS triggered elevated innate immune responses and glucose mobilization. However, our results that none of the pro-inflammatory cytokines were up-regulated due to continual MOS intake followed by LPS challenge (Tables 3 and S1, S2, S3) revealed that MOS counteracted LPS's detrimental effects on immunity. We also observed that energy demands of hosts fed MOS were sufficiently met by increased TCA cycle-derived energy. Contrastingly, VIRG failed to counteract or reduce LPS's inflammatory effects, as indicated by increased *IL-3* expression (Table 4). The higher energy demands of VIRG hosts necessitated glucose mobilization through intestinal gluconeogenesis and increased liver glycolytic activities. Based on these findings, we conclude that dietary MOS helped terminate LPS-induced inflammation earlier than VIRG. This beneficial effect of MOS may be explained by its inherent immune-stimulatory properties that caused mild immune stimulation, thereby ‘arming’ the body's defense mechanisms to rapidly and efficiently clear the endotoxin.

**Figure 4:**
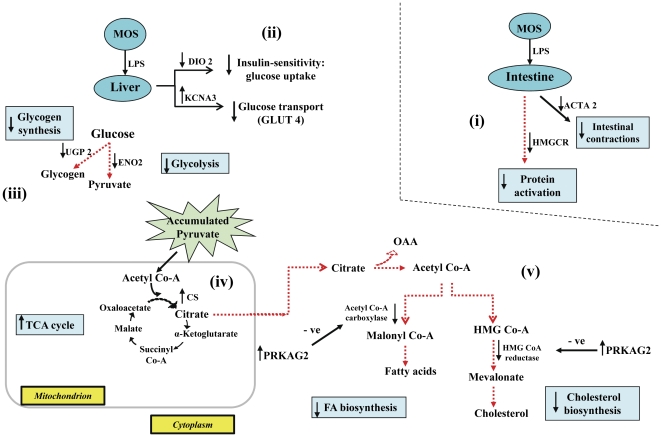
Schematic illustration of MOS effects on glucose metabolism between control and LPS-challenged hosts. (i) LPS triggered no major intestinal metabolic activities; (ii) in absence of glucose mobilization, liver glucose uptake and transport were repressed by *DIO2* down-regulation and *KCNA3* up-regulation, respectively; (iii) glycolysis and glycogen synthesis were coordinately reduced by *ENO2* and *UGP2* down-regulation, respectively; (iv) *CS* up-regulation increased TCA-derived energy from high liver pyruvate; (v) *PRKAG2* up-regulation inhibited fatty acid and cholesterol biosynthesis.

However, increased TCA activities surprisingly occurred among hosts fed MOS despite their reduced glycolytic activities. Although fatty acid and cholesterol synthesis genes are coordinately down-regulated during LPS-triggered systemic inflammation [47], we observed increased liver *de novo* fatty acid synthesis among hosts fed VIRG despite increased intestinal gluconeogenesis and liver glycolysis. Generally, fatty acid synthesis, which converts excess energy into energy reserves, occurs only when dietary carbohydrate intake exceeds immediate energy requirements. But, we observed a reduction in feed intake due to LPS challenge. Because inflammation is a dynamic biological immune reaction, molecular events at 24 h post-LPS challenge are a consequence of earlier immunological events. To help explain these apparently conflicting observations, we will briefly consider nutrient mobilization during intense inflammation. Whereas glycogenolysis and gluconeogenesis are frequently reported during intense inflammation, significant mobilization and catabolism of glucose may have significantly increased liver glucose and its glucose metabolites, including acetyl Co-A, pyruvate and citrate, levels in both MOS- and VIRG-fed hosts. In the absence of innate immune responses at 24 h post-LPS challenge, increased activity of CS, a key enzyme involved in TCA cycle, revealed that energy demands of MOS-fed hosts were mainly derived from liver glucose/glucose metabolites that accumulated earlier. Evidently, intestinal gluconeogenesis and liver glycolysis were not necessary and repressed (summarized in Figure 4).

**Figure 5:**
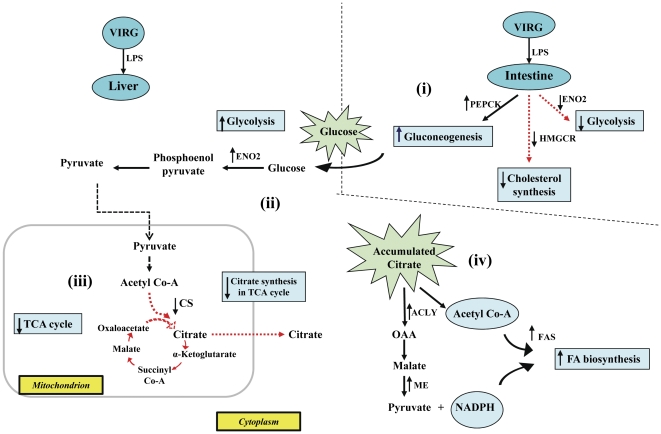
Schematic illustration of VIRG effects on glucose metabolism between control and LPS-challenged hosts. (i) LPS increased intestinal gluconeogenesis by up-regulating *PEPCK*; (ii) mobilized glucose increased liver glycolytic activities through *ENO2* up-regulation; (iii) *CS* down-regulation reduced utilization of glycolytic substrates by the TCA cycle for energy; (iv) *ACLY*, *ME* and *FAS* up-regulations increased liver fatty acid biosynthesis from high liver citrate.

In VIRG-fed hosts, however, elevated innate immune responses at 24 h post-LPS challenge required higher energy. Insufficient energy derived from accumulated liver glucose/glucose metabolites necessitated further glucose mobilization and catabolism through intestinal gluconeogenesis and liver glycolysis, respectively. However, as evidenced by *ACLY* up-regulation, exceptionally high liver citrate levels, which accumulated during intense inflammation, triggered *CS* down-regulation. Citrate is well recognized as a potent allosteric negative-feedback inhibitor of CS activity and plays a crucial role in liver metabolic activities. Evidently, to rapidly catabolize and deplete the accumulated liver cytosolic citrate after its efflux from the mitochondria where it is synthesized, (i) *ACLY* up-regulation generated high acetyl Co-A levels, (ii) *ME* up-regulation increased liver NADPH concentrations, (iii) whereas *FAS* up-regulation synthesized palmitate, the major fatty acid that ultimately yields long fatty acid chains, from acetyl Co-A, NADPH and malonyl Co-A, which is synthesized from acetyl Co-A by acetyl Co-A carboxylase (summarized in Figure 5). ACLY, ME and FAS are key lipogenic enzymes that convert liver cytoplasmic citrate into fatty acids. The preferential acetyl Co-A and NADPH utilization in *de novo* fatty acid biosynthesis mediated by *ACLY*, *ME* and *FAS* up-regulations is consistent with published reports [48]. In previously fasted and refed rats and chickens, increased liver lipogenesis was also mediated by *ACLY*, *ME* and *FAS* up-regulations [49], [50]. While increased glucose mobilization and decreased fatty acid synthesis have frequently been reported during intense inflammation [47], [51], here we report that the liver rapidly metabolized citrate into fatty acids to restore its citrate homoeostatic level during late inflammation in addition to glucose mobilization for body energy requirements.

**Figure 6:**
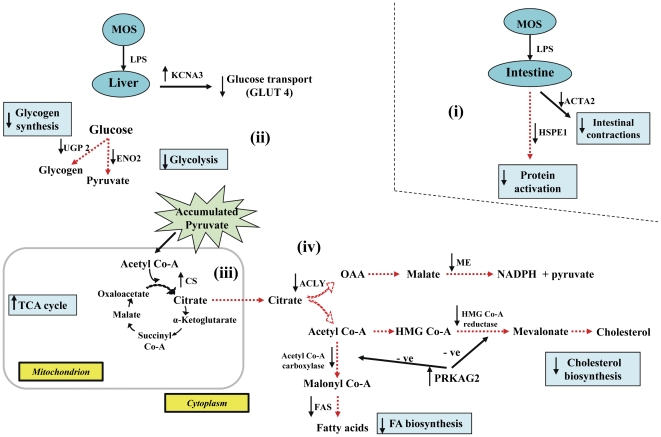
Schematic illustration of LPS effects on glucose metabolism between MOS- and VIRG-fed hosts. (i) LPS caused no major intestinal metabolic activities in MOS-fed hosts; (ii) in absence of liver glucose mobilization, *KCNA3* was up-regulated, whereas *ENO2* and *UGP2* down-regulation reduced glycolysis and glycogen synthesis, respectively; (iii) *CS* up-regulation increased TCA cycle-derived energy from high liver pyruvate; (iv) *ACLY*, *ME* and *FAS* down-regulations inhibited liver fatty acid biosynthesis; whereas PRKAG2 up-regulation inhibited fatty acid and cholesterol biosynthesis.

In agreement with O'Hea and Leveille [52], we observed that livers in chickens derived most of the NADPH required for fatty acid synthesis from the ME reaction, whereas livers in rats obtained about 65% of NADPH from the pentose phosphate pathway [53]. Collectively, these findings indicate that significantly more glucose was mobilized from the intestine and more glucose metabolites accumulated in the liver of VIRG hosts during the period of intense inflammation than MOS-fed hosts, and that VIRG failed to terminate innate immune responses earlier. But, when challenged with LPS, we observed an elevation in innate immune responses, principally mediated by intestinal IL-3, among hosts fed MOS than VIRG. Although these results were not surprising considering the additive immune-stimulatory effects of MOS and LPS, no major nutrient mobilization processes occurred among LPS-challenged hosts fed MOS (summarized in Figure 6). TCA-derived energy from high liver glucose and glucose metabolites which accumulated earlier than 24 h of LPS challenge was sufficient to meet energy demands of the hosts fed MOS.

Livers and kidneys are well-recognized gluconeogenic organs in humans and mice [54], [55]. Whereas the intestine is equivocally reported as a gluconeogenic organ in mice [56], [57], we are among the first to demonstrate that the chicken small intestine, but not skeletal muscles, is also a gluconeogenic organ that was regulated by *PEPCK*. We have discussed the increased intestinal gluconeogenesis at 24 h post-LPS challenge. Given that MOS and VIRG are not absorbed across the intestinal epithelium, these macromolecules produce localized effects in the intestines. Therefore, all our findings evidenced cross talks between intestinal mucosal immunity and systemic immunity. This is the first study demonstrating that MOS can beneficially modulate innate immunity and nutrient metabolism during late systemic inflammation.

In summary, late inflammation was principally modulated by IL-3. In contrast to antibiotics like VIRG, MOS elicited several beneficial responses: (i) terminated *Salmonella* LPS-induced systemic inflammation earlier, presumably due to its inherent intestinal innate immune-stimulatory properties; and (ii) reduced the magnitude of glucose mobilization. Therefore, this study potentiates the use of natural immuno-modulators, such as a MOS, to attenuate *Salmonella*-induced systemic inflammation both among human and animal hosts, and without posing the risk of antibiotic-resistance development.

## Materials and Methods

### Chickens, experimental diets and LPS challenge

Hatched chicks (Cobb 500 broilers) were raised in two groups (n = 64/group). In each bird group (8 cages/diet), half was fed a diet containing MOS (2 kg/ton BioMos®; Alltech Inc., Nicholasville, KY) or virginiamycin (16.5 mg/kg), as described [46]. To induce an acute inflammatory response, group 1 hosts (n = 64) were injected i.p. with 3 ml of *Salmonella* Typhimurium LPS (100 mg LPS/L, Sigma-Aldrich, ON, Canada) whereas group 2 control hosts were saline-injected at 14 d of age. All animal procedures were approved by the McGill Animal Care Committee (protocol number 5399). All hosts had free access to feed and water.

### Bodyweight, feed intake and body temperature measurements

All non-challenged control (saline-injected) and LPS-challenged hosts were individually weighed at 0 (initial BW), 12, 24 and 48 h post-injection to determine BW gain relative to initial BW. Average feed consumption of chickens was calculated at similar time points. Body temperatures were recorded after 0, 2, 4, 6, 8, 12, 24 and 48 h of saline or LPS injection using a thermocouple rectal probe thermometer (Physitemp Instruments Inc., Clifton, NJ).

### Liver weights and tissue samples collection

Chickens (n = 8/diet/group) were randomly euthanized at 12, 24 and 48 h post-injection and liver weights of respective chickens were expressed relative to their final BW. At 24 h after saline and LPS injections, liver, intestine (jejunum) and skeletal muscle (breast meat) samples (n = 6 /diet/group) were immediately snap frozen in liquid nitrogen, and stored at −80°C for later RNA extraction.

### Microarray analysis

After 24 h, total RNA was extracted from liver, intestine (jejunum) and skeletal muscle (breast) tissues using Trizol reagent and Purelink RNA Mini Kit (Invitrogen). Isolated total RNA was quantified on the basis of its absorption at 260 nm using a Nanodrop® ND-1000 spectrophotometer (NanoDrop Technologies, Wilmington, DE), and visualized on an agarose gel to check quality. RNA was retrotranscribed into Cy3 or Cy5 aminoallyl labelled cDNA and hybridized onto chicken-specific focused oligonucleotides microarrays. The microarray platform used (accession number GPL13457) and data files (accession number GSE28959) are registered at the MIAME compliant National Centre for Biotechnology Information (NCBI) Gene Expression Omnibus (GEO) archive (http://ncbi.nlm.nih.gov/projects/geo). Briefly, 70mers chicken-specific oligonucleotides, obtained from Operon Biotechnologies Inc. (Germantown, MD), were spot printed on UltraGAPS Amino-Silane Coated Slides (Corning Inc., Acton, MA) as described [58]. Each oligonucleotide sequence (probe) was replicated 12 times per array.

### cDNA labeling and microarray hybridization

A total of 12 microarrays was used per tissue and chicken group (n = 6/diet) in a 2×2 factorial design and complete interwoven loop arrangement (Figure S5; [59]). First, RNA was retrotranscribed into aminoallyl labelled cDNA using the ChipShot Indirect Labelling and Clean-Up System Kit (Promega, Madison, WI) and Cy3 or Cy5 fluorescent dye (Amersham Biosciences Corp., Piscataway, NJ) according to the manufacturer's recommendations. Reverse transcription was carried out at 42°C for 2 h, followed by RNase H digestion for 15 min at 37°C. Briefly, a reactive amine derivative of 5-(3-aminoallyl)-2′-deoxyuridine 5′-triphosphate was incorporated during reverse transcription. Subsequent to reverse transcriptase reaction, succinimidyl esters of Cy3 or Cy5 were covalently bonded to aminoallyl-labelled cDNAs. Cy-3 and Cy-5 labelled cDNA were then purified, combined and hybridized to the array for 24 h in darkness by making use of the Pronto Plus! Microarray Hybridisation Kit (Corning Inc., NY).

### Microarray data analysis

Hybridized arrays were scanned twice at 65% (Cy3) and 50% (Cy5) laser power using a ScanArray GX PLUS Microarray Scanner (PerkinElmer Life and Analytical Sciences, Shelton, CT) to obtain Cy3∶Cy5 intensity ratios of labelled cDNA hybridized to complementary oligonucleotide sequences on the array. Spot intensity data were extracted using the ScanAlyze Software (Standford University, Standford, CA) and analyzed using the JMP Genomics software (SAS Institute Inc., Cary, NC). Data were log_2_ transformed prior to normalization by using locally weighted regression and smoothing, first within array (ratio analysis) and then across arrays (Lowess normalization). Normalized data were monitored by distribution analysis of the transformed data. Finally, the normalized log_2_ transformed data were analyzed using a two-way ANOVA, as described [60]. Expression values were modeled as: Y_ijklm_ = μ+A_i_+C_j_+D_k_+I_l_+DI_kl_+e_ijklm_, where μ represents the overall mean value, A_i_: random effect for arrays (i = 1, 2…‥12), C_j_: main effect of Cy-dye (j = Cy-3 or Cy-5), D_k_: main effect of diet (k = MOS or VIRG), I_l_: main effect of injection (l = saline or LPS), and DI_kl_: interaction effect between diet and injection, and e_ijklm_: random error. Mean intensities were tested using the false discovery rate (FDR) multiple comparison *t* test and differentially expressed genes were declared at *P*<0.05. Finally, for each pairwise comparison, significantly different genes were filtered based on their mean intensity values to determine up- or down-regulated genes due to diet, injection and diet*injection.

### Real-time quantitative PCR analysis

Real-time quantitative PCR (RT-qPCR) was used for validation of differential gene expressions observed in microarrays. Total RNA was retrotranscribed into cDNA by using 1 µg total RNA and iScript cDNA Synthesis Kit (Bio-Rad, ON, Canada), following the manufacturer's instructions. RT-qPCR was performed using the Bio-Rad CFX384 RT-qPCR Detection System, SsoFast Evagreen Supermix (Bio-Rad) and primer-set sequences (Table 6). RT-qPCR reactions were performed at 95°C for 5 min, followed by 39 cycles of 95°C for 15 s and 60°C for 30 s. A melting curve program was included at the end of each RT-qPCR to verify presence of a unique product. Relative intestinal (*PEPCK*) and liver (*ACLY*, *ME*, *CS* and *FAS*) gene expression levels were normalized to *GAPDH* or *beta-2 microglobulin*, respectively. The expression stability of the reference genes were tested using the geNorm software (available at: http://medgen.ugent.be/~jvdesomp/genorm/). Samples were analyzed in technical duplicates, and differential gene expressions were determined using the comparative standard curve method.

**Table 6:** Primers-set sequences used to analyze gene expression by quantitative PCR.

Gene	Forward primer^a^	Reverse primer^a^	Amplicon length (bp)	PubMed Accession No.
PEPCK	CTGCTGGTGTGCCTCTTGTA	TTCCCTTGGCTGTCTTTCC	259	NM_205471
ACLY	GGCGTGAATGAACTGGCTAAC	TAGTCTTGGCATAGTCATAGGTCTGTTG	79	NM_001030540
ME	TGCCAGCATTACGGTTTAGC	CCATTCCATAACAGCCAAGGTC	175	NM_204303
FAS	TGAAGGACCTTATCGCATTGC	GCATGGGAAGCATTTTGTTGT	96	NM_205155
GAPDH	TGCCATCACAGCCACACAGAAG	ACTTTCCCCACAGCCTTAGCAG	123	NM_204305
Beta-2 microglobulin	AAGGAGCCGCAGGTCTA	CTTGCTCTTTGCCGTCATAC	151	Z48921

a Sequences are indicated from 5′ end to 3′ end of oligonucleotides.

### Liver metabolites measurements

Liver citrate and pyruvate levels were measured by specific enzymatic reactions using the Citrate and Pyruvate Assay Kits (BioVision, CA, USA), following the manufacturer's instructions with few modifications. Briefly, 0.5 g of liver tissues were homogenized completely by sonication in 700 µL of respective buffer solutions and then centrifuged at 15,000 g for 10 mins to remove cell debris. After the supernatant was deproteinized using the Deproteinizing Sample Preparation Kit (BioVision), a 100 µL sample volume was used for analysis. Reaction mix was prepared without buffer dilution.

### Statistical analysis

Except for microarray data, all data were analyzed as a two-way ANOVA and a 2×2 factorial arrangement to determine the main effects of diet and injection, and their interaction effects by using the MIXED procedure of SAS (SAS Institute, 2003). For bodyweight, liver weight and body temperature data, a Nested Model Design was also employed with cages nested within diet*injection, as follows: Y_ijkl_ = μ+Diet_i_+Injection_j_+Diet_i_*Injection_j_+Cage_ijk_+e_ijkl_, where μ represents the overall mean value, Diet_i_: fixed effect of diet (i = MOS or VIRG), Injection_j_: fixed effect of injection (j = saline or LPS), Diet_i_ * Injection_j_: interaction effect between diet and injection, Cage_ijk_: random effect of cage nested within diet*injection (k = 1,2,…8), and e_ijkl_: random error. Differences among treatment means were tested using Scheffe's *t* test and statistical significance declared at *P*<0.05.

## Supporting Information

Figure S1 Innate immune-stimulatory effects of LPS caused elevation in body temperatures. Antibiotic (VIRG)- and MOS-fed hosts were injected i.p. with LPS. At 0, 2 and 48 h after injection, body temperatures were not different between LPS-challenged and non-challenged control (saline) hosts (*A*), or between VIRG- and MOS-fed hosts within the LPS-challenged or control group, respectively (*B*), or between hosts fed VIRG and MOS (*C*). However, in comparison with control hosts, LPS significantly increased (*P*<0.05) body temperatures at 4, 6, 8, 12 and 24 h post-injection (*A*). But, such increase in body temperatures were not observed between VIRG and MOS hosts within the LPS-challenged or control group, respectively (*B*), or between VIRG- and MOS-fed hosts irrespective of injection type (*C*). Results are expressed as mean ± SEM. Supercripts: (a,b) denote statistical differences among treatment means at a particular time point, *P*<0.05, Scheffe's multi-comparison *t*-test.
(PDF)

Figure S2 The effects of LPS injected i.p. on feed intake in antibiotic- (VIRG) and MOS-fed hosts. LPS significantly reduced feed intake (*P*<0.05) at 12 h post-injection (*A*). However, feed intake was not different between hosts fed the VIRG and MOS diet (*B*), or between VIRG and MOS hosts within the LPS-challenged or non-challenged control (saline) group, respectively (*C*). At 24 and 48 h post-injection, feed intake was not different between LPS and control hosts (*A*), hosts fed the VIRG and MOS diet (*B*), or between VIRG and MOS hosts within the LPS-challenged or control group, respectively (*C*). Results are expressed as mean ± SEM. Supercripts: (a,b) denote statistical differences among treatment means at a particular time, *P*<0.05, Scheffe's multi-comparison *t*-test.
(PDF)

Figure S3 The effects of LPS injected i.p. on bodyweight (BW) gain in antibiotic- (VIRG) and MOS fed hosts. LPS significantly reduced BW (*P*<0.05) after 12, 24 and 48 h of injection (*A*). In contrast to MOS-fed hosts, those fed VIRG grew faster (*P*<0.05) at 48 h only (*B*). But, BW gain was not different between VIRG and MOS hosts within the LPS-challenged or non-challenged control (saline) group, respectively (*C*). Results are expressed as mean ± SEM. Supercripts: (a,b) denote statistical differences among treatment means at a particular time point, *P*<0.05, Scheffe's multi-comparison *t*-test.
(PDF)

Figure S4 The effects of LPS injected i.p. on liver weights of antibiotic- (VIRG) and MOS fed hosts. LPS significantly increased (*P*<0.05) liver weights at 12 and 24 h post-injection, but not after 48 h (*A*). However, at all times, liver weights were not different between hosts fed the VIRG and MOS diet (*B*), or between VIRG and MOS hosts within the LPS-challenged or non-challenged control (saline) group, respectively (*C*). Results are expressed as mean ± SEM. Supercripts: (a,b) denote statistical differences among treatment means at a particular time, *P*<0.05, Scheffe's multi-comparison *t*-test.
(PDF)

Figure S5 Schematic of the 2×2 factorial experimental design in an interwoven loop arrangement for each tissue (liver, intestine or skeletal muscles). Diet×Injection is denoted as VC (VIRG×non-challenged control hosts), VL (VIRG×LPS-challenged hosts), BC (MOS×non-challenged control hosts), and BL (MOS×LPS-challenged hosts). Each arrow represents an array (total: 12) consisting of 2 aminoallyl labelled cDNA, either Cy-3 or Cy-5.
(PDF)

Table S1 Fold change and P values of gene expressions due to prebiotic and antibiotic in intestinal tissues of non-challenged control and LPS-challenged birds.
(XLSX)

Table S2 Fold change and P values of gene expressions due to prebiotic and antibiotic in liver tissues of non-challenged control and LPS-challenged birds.
(XLSX)

Table S3 Fold change and P values of gene expressions due to prebiotic and antibiotic in skeletal muscle tissues of non-challenged control and LPS-challenged birds.
(XLSX)
