# Supplementary figures and images for: Cell Walls of Saccharomyces cerevisiae Differentially Modulated Innate Immunity and Glucose Metabolism during Late Systemic Inflammation
Source: PLoS One. 2012 Jan 17;7(1):e30323. doi: 10.1371/journal.pone.0030323 (PMC3260269; doi:10.1371/journal.pone.0030323)

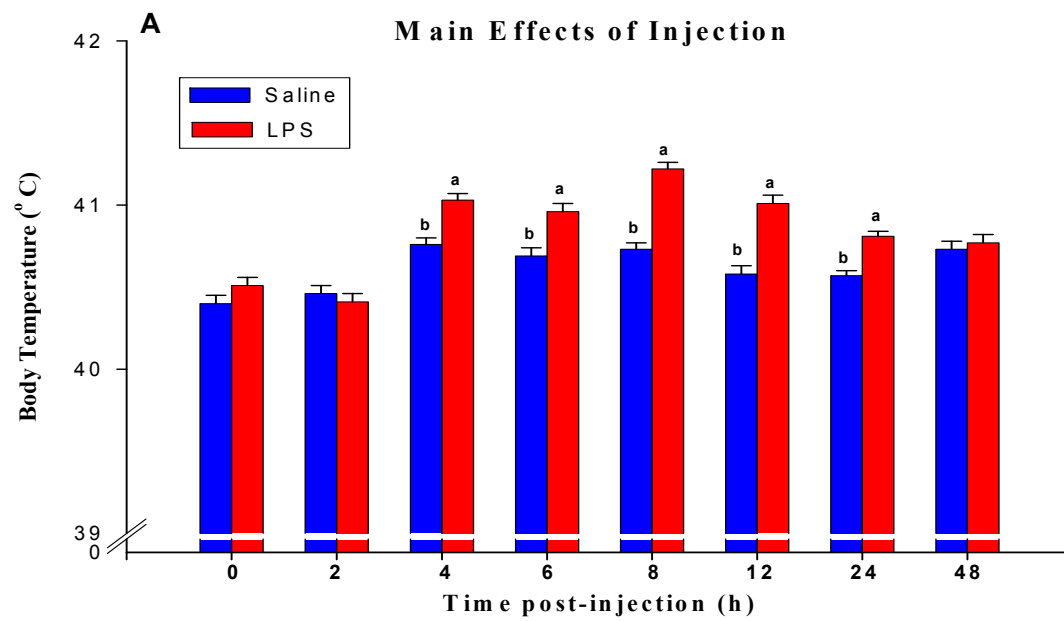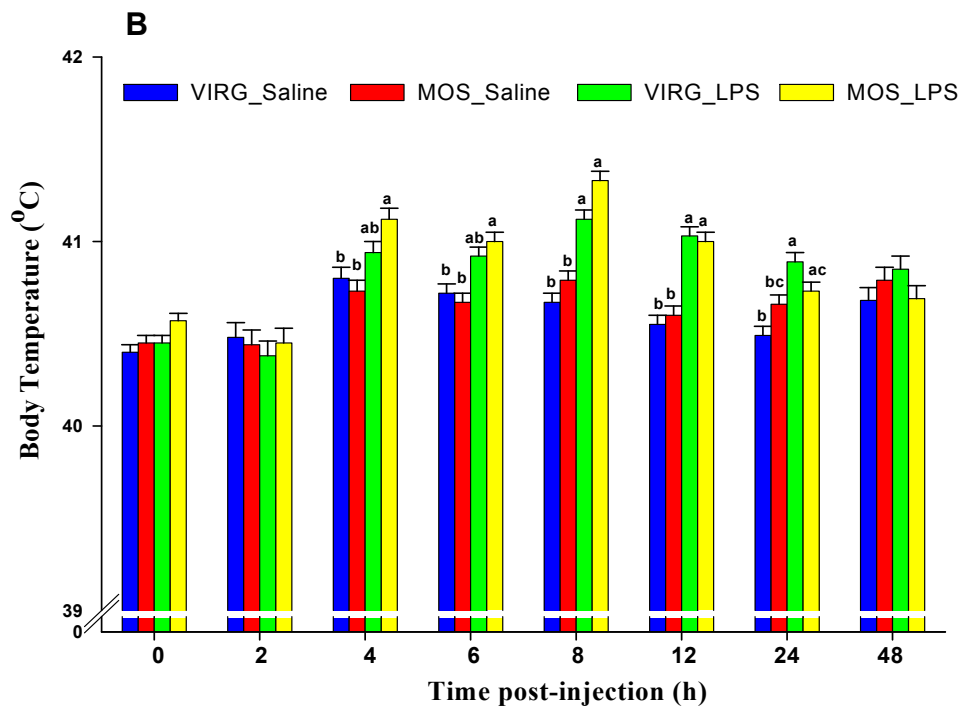

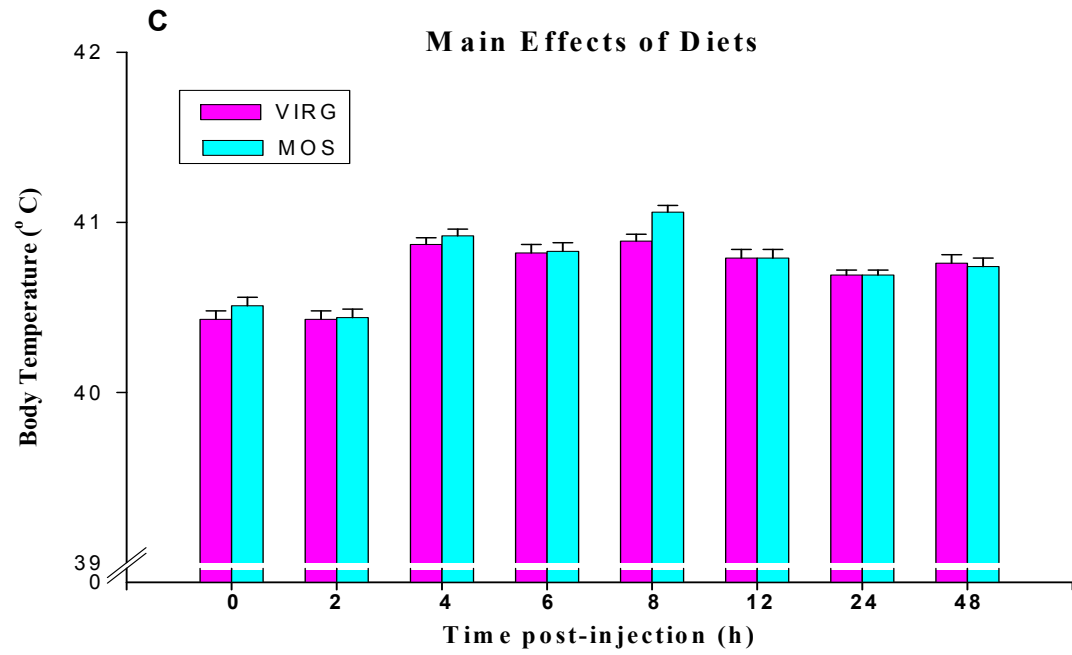

Supplement: Figure S1 — Innate immune-stimulatory effects of LPS caused elevation in body temperatures. Antibiotic (VIRG)- and MOS-fed hosts were injected i.p. with LPS. At 0, 2 and 48 h after injection, body temperatures were not different between LPS-challenged and non-challenged control (saline) hosts (A), or between VIRG- and MOS-fed hosts within the LPS-challenged or control group, respectively (B), or between hosts fed VIRG and MOS (C). However, in comparison with control hosts, LPS significantly increased (P<0.05) body temperatures at 4, 6, 8, 12 and 24 h post-injection (A). But, such increase in body temperatures were not observed between VIRG and MOS hosts within the LPS-challenged or control group, respectively (B), or between VIRG- and MOS-fed hosts irrespective of injection type (C). Results are expressed as mean ± SEM. Supercripts: (a,b) denote statistical differences among treatment means at a particular time point, P<0.05, Scheffe's multi-comparison t-test. (PDF) [file pone.0030323.s001.pdf]

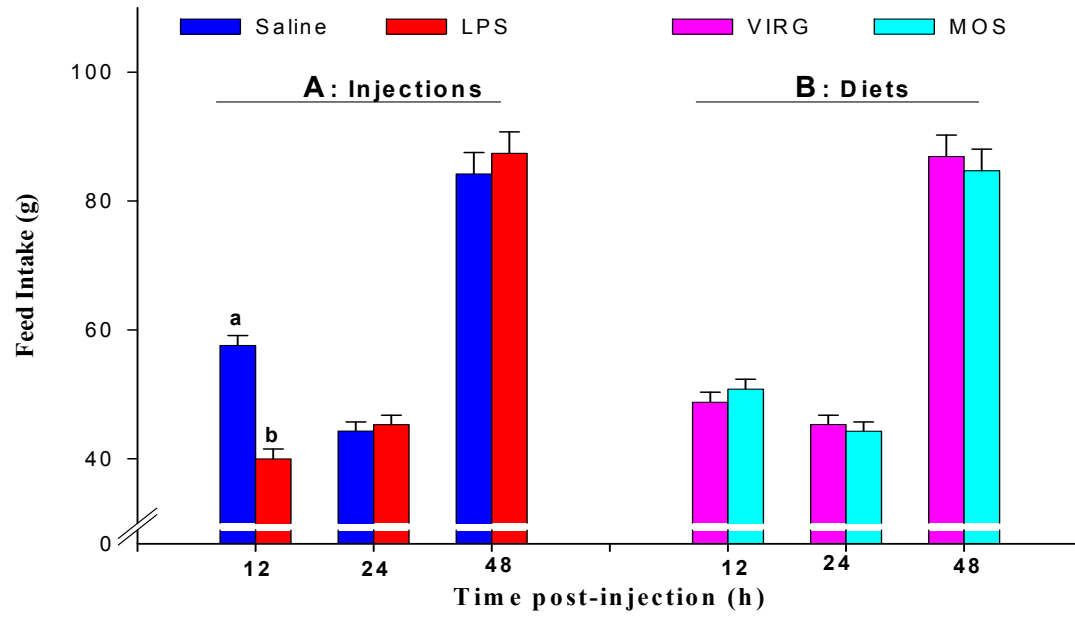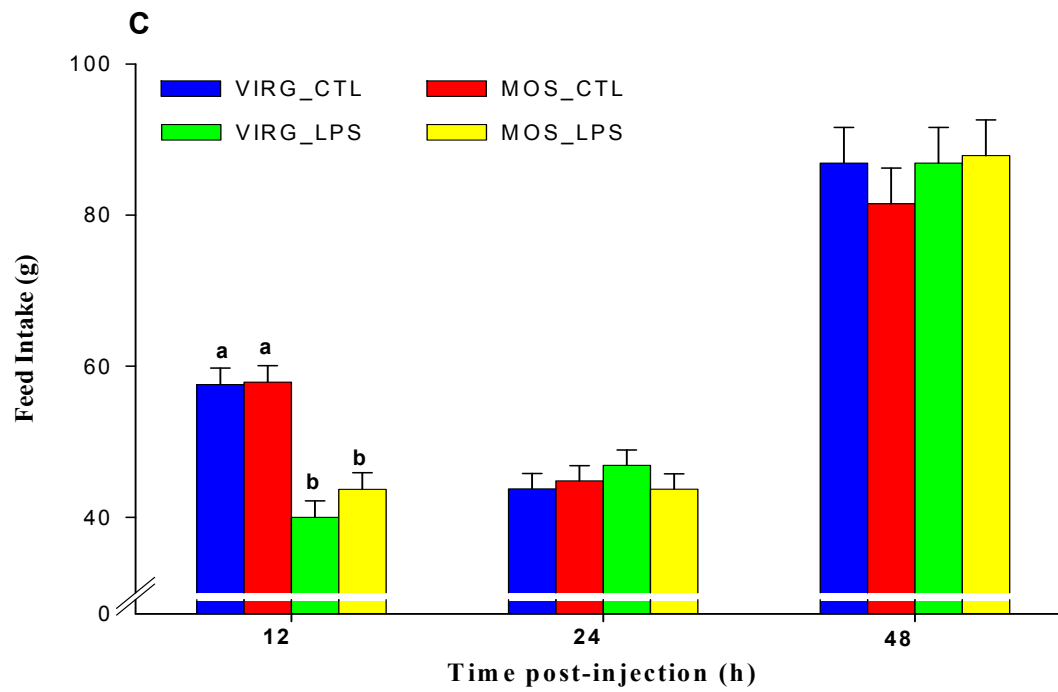

Supplement: Figure S2 — The effects of LPS injected i.p. on feed intake in antibiotic- (VIRG) and MOS-fed hosts. LPS significantly reduced feed intake (P<0.05) at 12 h post-injection (A). However, feed intake was not different between hosts fed the VIRG and MOS diet (B), or between VIRG and MOS hosts within the LPS-challenged or non-challenged control (saline) group, respectively (C). At 24 and 48 h post-injection, feed intake was not different between LPS and control hosts (A), hosts fed the VIRG and MOS diet (B), or between VIRG and MOS hosts within the LPS-challenged or control group, respectively (C). Results are expressed as mean ± SEM. Supercripts: (a,b) denote statistical differences among treatment means at a particular time, P<0.05, Scheffe's multi-comparison t-test. (PDF) [file pone.0030323.s002.pdf]

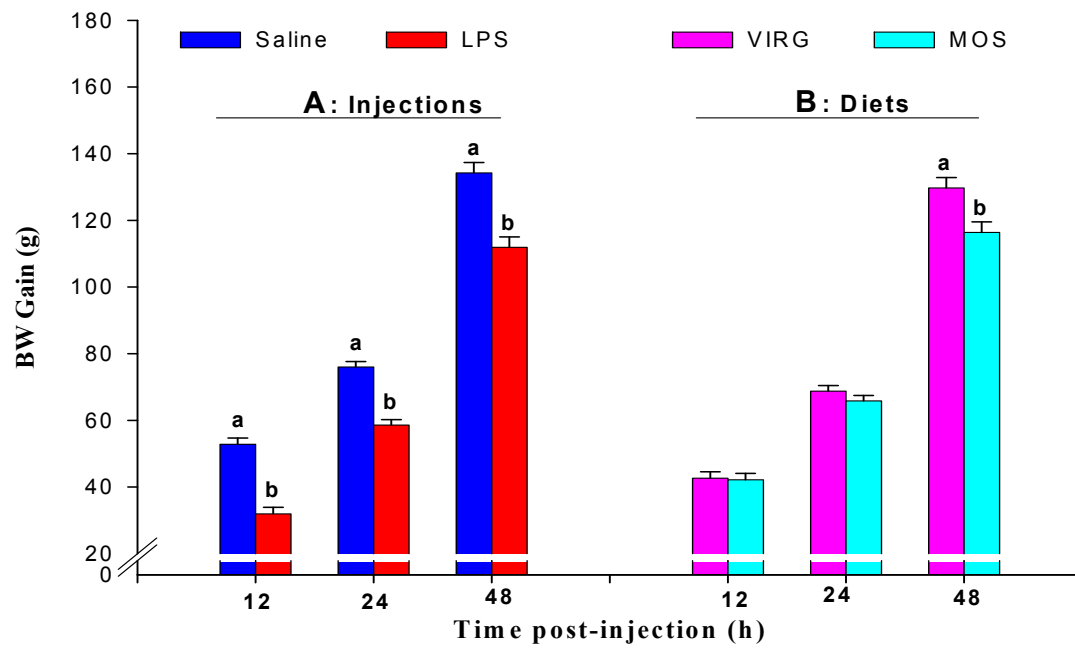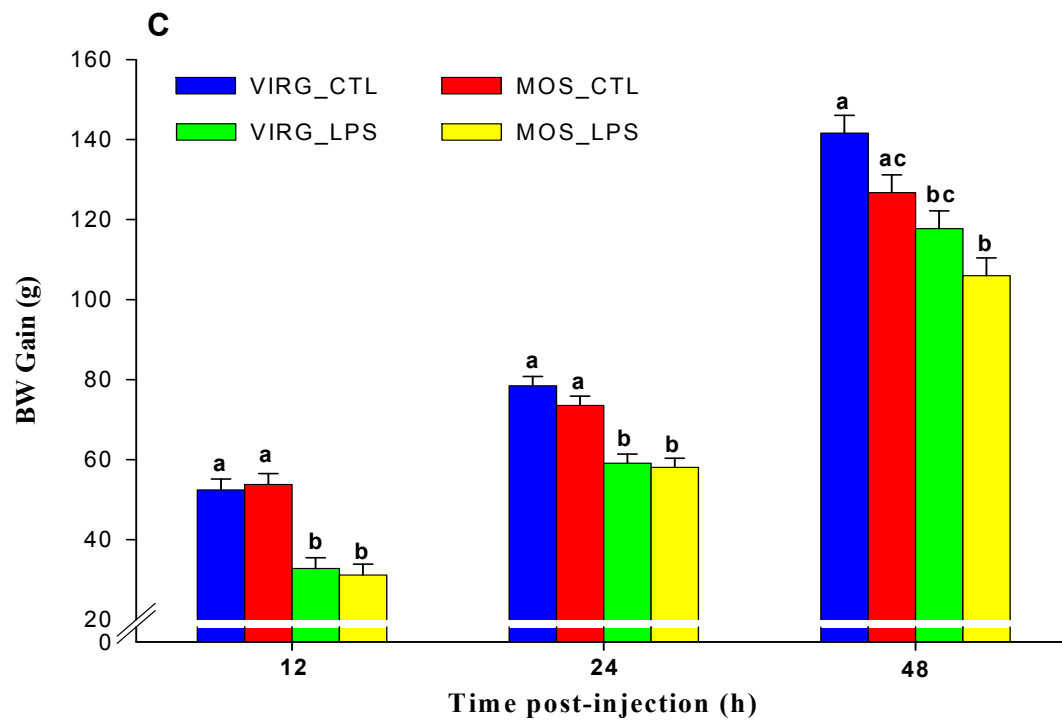

Supplement: Figure S3 — The effects of LPS injected i.p. on bodyweight (BW) gain in antibiotic- (VIRG) and MOS fed hosts. LPS significantly reduced BW (P<0.05) after 12, 24 and 48 h of injection (A). In contrast to MOS-fed hosts, those fed VIRG grew faster (P<0.05) at 48 h only (B). But, BW gain was not different between VIRG and MOS hosts within the LPS-challenged or non-challenged control (saline) group, respectively (C). Results are expressed as mean ± SEM. Supercripts: (a,b) denote statistical differences among treatment means at a particular time point, P<0.05, Scheffe's multi-comparison t-test. (PDF) [file pone.0030323.s003.pdf]

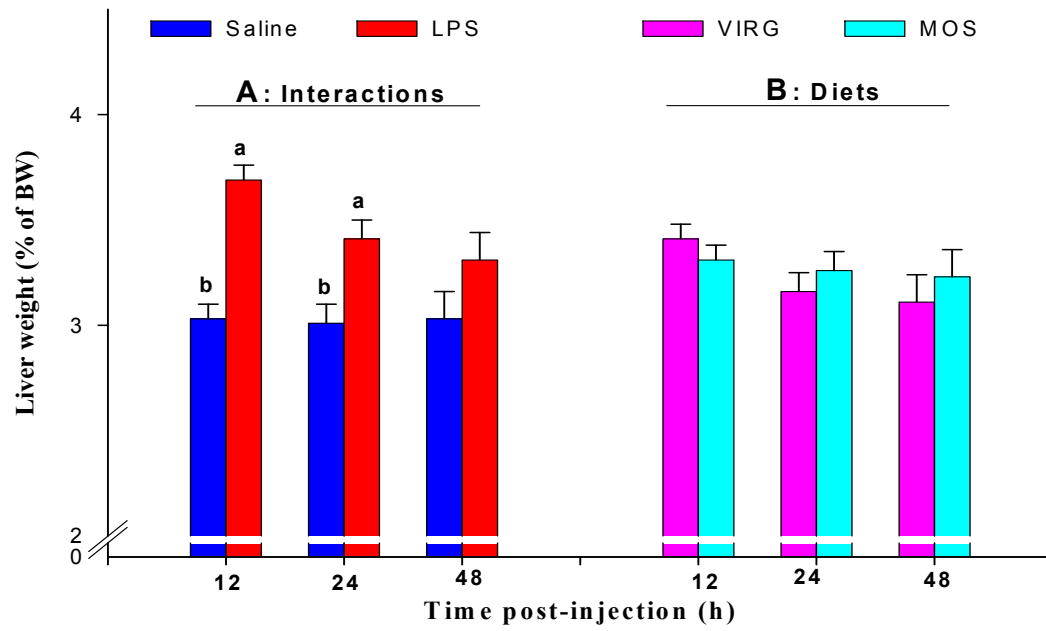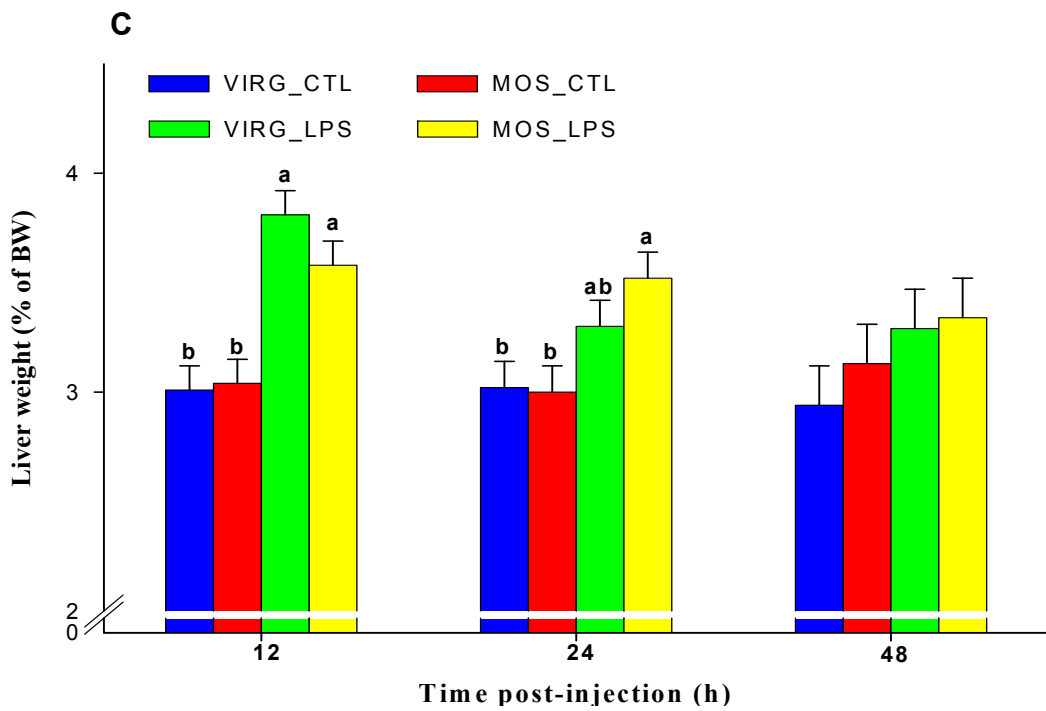

Supplement: Figure S4 — The effects of LPS injected i.p. on liver weights of antibiotic- (VIRG) and MOS fed hosts. LPS significantly increased (P<0.05) liver weights at 12 and 24 h post-injection, but not after 48 h (A). However, at all times, liver weights were not different between hosts fed the VIRG and MOS diet (B), or between VIRG and MOS hosts within the LPS-challenged or non-challenged control (saline) group, respectively (C). Results are expressed as mean ± SEM. Supercripts: (a,b) denote statistical differences among treatment means at a particular time, P<0.05, Scheffe's multi-comparison t-test. (PDF) [file pone.0030323.s004.pdf]

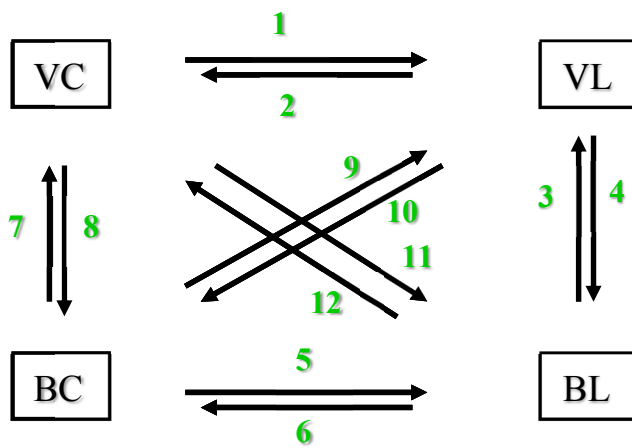

Supplement: Figure S5 — Schematic of the 2×2 factorial experimental design in an interwoven loop arrangement for each tissue (liver, intestine or skeletal muscles). Diet×Injection is denoted as VC (VIRG×non-challenged control hosts), VL (VIRG×LPS-challenged hosts), BC (MOS×non-challenged control hosts), and BL (MOS×LPS-challenged hosts). Each arrow represents an array (total: 12) consisting of 2 aminoallyl labelled cDNA, either Cy-3 or Cy-5. (PDF) [file pone.0030323.s005.pdf]
